# Inventory of oncologists’ unmet needs for tools to support decision-making about palliative treatment for metastatic colorectal cancer

**DOI:** 10.1186/s12911-018-0712-9

**Published:** 2018-12-14

**Authors:** Ellen G. Engelhardt, Dóra Révész, Hans J. Tamminga, Cornelis J. A. Punt, Miriam Koopman, Bregje D. Onwuteaka-Philipsen, Ewout W. Steyerberg, Henrica C. W. de Vet, Veerle M. H. Coupé

**Affiliations:** 1 Department of Epidemiology and Biostatistics, Amsterdam UMC, location VUMC, F-wing Medical Faculty building, PO Box 7057 1007, MB Amsterdam, The Netherlands; 2 Department of Medical Oncology, Amsterdam UMC, location AMC, Amsterdam, The Netherlands; 3 Department of Medical Oncology, University Medical Center Utrecht and University Utrecht, Utrecht, The Netherlands; 4 Department of Public and Occupational Health, and Palliative Care Expertise Centre, VU University Medical Center, Amsterdam Public Health Research Institute, Amsterdam, The Netherlands; 5 Department of Public Health, Centre for Medical Decision Making, Erasmus Medical Center, Rotterdam, The Netherlands and Department of Medical Statistics, Leiden University Medical Center, Leiden, The Netherlands

**Keywords:** Metastatic colorectal cancer, Decision support tools, Questionnaires

## Abstract

**Background:**

Decision-making about palliative care for metastatic colorectal cancer (mCRC) consists of many different treatment-related decisions, and there generally is no best treatment option. Decision support systems (DSS), e.g., prognostic calculators, can aid oncologists’ decision-making. DSS that contain features tailored to the needs of oncologists are more likely to be implemented in clinical practice. Therefore, our aim is to inventory colorectal cancer specialists’ unmet decision support needs.

**Methods:**

We asked oncologists from the Dutch colorectal cancer group (DCCG), to participate in an online inventory questionnaire on their unmet decision support needs. To get more in-depth insight in required features of the DSS they need, we also conducted semi-structured telephone interviews.

**Results:**

Forty-one oncologists started the inventory questionnaire, and 27 of them completed all items. Of all respondents, 18 were surgeons (44%), 22 were medical oncologists (54%), and 28 (68%) had more than 10 years of experience treating mCRC. In both the inventory questionnaire and interviews, respondents expressed a need for an overarching DSS incorporating multiple treatment options, and presenting both the treatment benefits and harms. Respondents found it relevant for other outcomes, such as cost-effectiveness of treatment or quality of life, to be incorporated in DSS. There was also a wish for DSS incorporating an up-to-date “personalized” overview of the ongoing trials for which a specific patient is eligible.

**Conclusions:**

Experienced oncologists indicate that their treatment advice is currently almost solely based on the available clinical guidelines. They experience a lack of good quality DSS to help them personalize their treatment advice. New tools integrating multiple treatment options and providing a broad range of clinically relevant outcomes are urgently needed to stimulate and safeguard more personalized treatment decision-making.

## Background

Clinical decision-making about palliative treatment for metastatic colorectal cancer (mCRC) is complex. The patient population is heterogeneous in terms of type of metastases and prognosis, and decision-making encompasses many decisions during the course of treatment. The key premise of palliative treatment is to maintain and/or improve patients’ quality of life. Clinical guidelines give treatment options for the specific stages of disease and are of a general nature. In this era of personalized treatment, decision support systems (DSS), e.g., online prognostic calculators, decision trees and nomograms, can help oncologists to better conceptualize the trade-off between treatment benefits and harms for individual patients. These tools can thereby help them to give a more personalized evidence-based treatment advice to their patients, and if adequately formatted DSS could be used during consultations to inform patients.

Although DSS can be helpful, their usefulness depends on their quality, whether their development was methodologically sound, whether they have been adequately externally validated, and which predictors their estimates are based on. Currently, there is a limited number of DSS available for the mCRC setting [1–11]. The majority of available tools have not been externally validated, and for none of them a clinical impact analysis has been performed [12]. Also, available DSS are mainly based on patient, tumor, and treatment characteristics, and rarely include genetic markers. Research into clinically important genetic markers to aid further personalization of treatment decision-making is currently ongoing. There is some evidence that oncogenes (e.g., *KRAS*, *BRAF* or *PIK3CA*), and tumor suppressor genes (e.g., *APC*, *TP53* or *PTEN*) yield clinically relevant information that can help oncologists select patients who will benefit from specific (targeted) treatments [13–17]. However, the available data is not yet at a stage that these markers can be used in clinical practice. Further, if DSS only provide information about the treatment benefits, this can shift the focus away from the potential side-effects, and could thus impede adequate valuation of the trade-off between the treatment benefits and harms. Thus, DSS should incorporate information on both the benefits and harms of treatment.

Good quality DSS to aid decision-making about palliative treatment for mCRC are not yet available. It begs the question whether gastroenterologists, surgical and medical oncologists have a need for decision support tools. Therefore, we disseminated an online questionnaire to inventory colorectal cancer specialists’ need for new DSS, and gain insight into what features such new tools should have in order to meet the existing clinical needs.

## Methods

### Online inventory questionnaire development and dissemination

In the inventory questionnaire, colorectal cancer specialists (further labeled as oncologists) were asked to indicate whether or not they have unmet decision support needs, and if this was the case, they were asked to describe the requirements and characteristics of new DSS they need using an open-ended question. With the inventory questionnaire we also assessed respondent characteristics, namely a) type of hospital they work at, b) number of years of experience treating patients with mCRC, c) number of mCRC patients treated on a yearly basis, d) which DSS for palliative treatment decisions in patients with mCRC they are familiar with and utilize, and e) which factors oncologists consider in order to reach their treatment advise. All questions pertaining to respondent characteristics were multiple choice including the option ‘other’, with space to give another answer. The inventory questionnaire was developed by the research team that included clinical (CJAP) and palliative care (BDOP) experts.

A link to the online inventory questionnaire was sent to surgical, radiation, and medical oncologists who are members of the Dutch colorectal cancer group (DCCG; *N* = 360 members) via the DCCG secretariat. DCCG-members are experts in the field of colorectal cancer (CRC) diagnosis and treatment. The DCCG is a working party in which healthcare professionals from across the Netherlands specializing in colorectal cancer take part. Participants’ did not receive compensation for participation. Three weeks after the original invitation to participate, a reminder was sent.

### Individual interviews

With the inventory questionnaire we were unable to delve deeply into respondents’ existing unmet decision support needs. Therefore, we also conducted semi-structured individual telephone interviews. The interviewer had a topic list (i.e., use of DSS, clinical situation for which DSS are useful, unmet decision support needs) that needed to be addressed, but any other topics raised by the interviewee would also be explored. Respondents provided us with contact information in the inventory questionnaire if they were willing to participate in these interviews. All interviews were audiotaped.

### Data analyses

Descriptive analyses were performed on the inventory questionnaire data. One of the in total 42 respondents indicated not to have any experience with palliative care for mCRC, and was excluded from all analyses. In order to identify key points in the audio recordings of the interviews, the interviewer (EGE) performed a thematic assessment of all answers. Themes from the interviews were integrated with information from the online inventory questionnaires to formulate the unmet needs.

## Results

### Population characteristics

Forty-one oncologists with experience treating mCRC started the inventory questionnaire and 27 of them completed it. Respondents were mainly medical oncologists (*n* = 22) and surgeons (*n* = 18) working at general teaching hospitals (*n* = 25), and general hospitals (*n* = 10) (Table 1). Of the oncologists, 68% had more than 10 years of experience treating patients with incurable mCRC, and 57% saw between 11 and 50 incurable mCRC patients per year. Four out of the nine oncologists who indicated in the inventory questionnaire that they would be willing to be interviewed could be reached for an interview. Three of them were medical oncologists, and one was a surgeon, and they regularly treated patients with incurable mCRC.

**Table 1:** Characteristics of participants

	Respondents inventory questionnaire*N* = 41 (100%)
Medical specialty	
Surgeon	18 (44)
Medical oncologist	22 (54)
Gastroenterologist	1 (2)
Type of hospital	
General hospital	9 (22)
General teaching hospital	25 (61)
University medical center	6 (15)
Specialized oncology hospital	1 (2)
Experience treating patients with incurable CRC	
≤ 2 years	1 (2)
3–5 years	6 (15)
6–10 years	6 (15)
> 10 years	28 (68)
Number of patients treated on a yearly basis	
0–5 patients	3 (7)
6–10 patients	6 (15)
11–20 patients	15 (37)
21–50 patients	9 (22)
> 50 patients	8 (20)

**Fig. 1:**
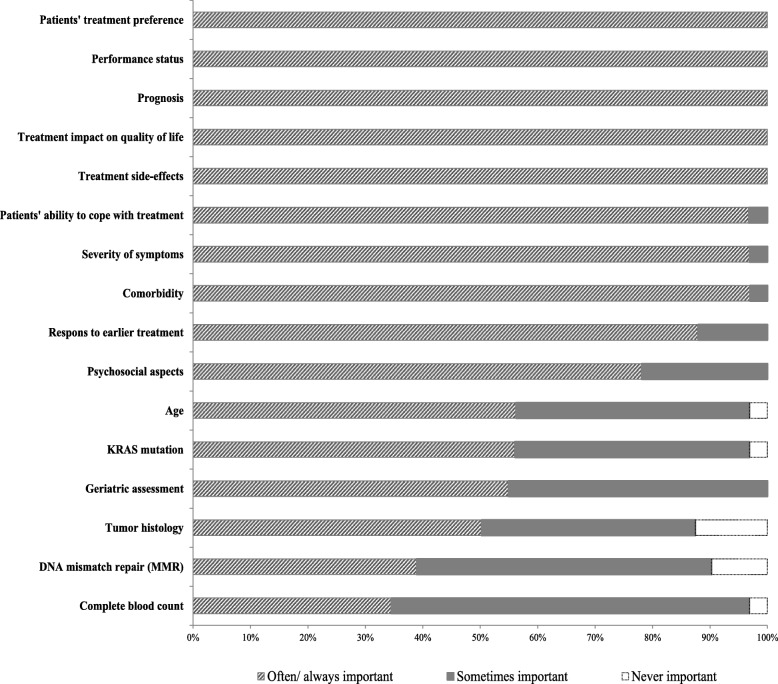
Overview of factors considered by medical specialists to inform their mCRC treatment advice (*N* = 32)

In the questionnaire almost all respondents indicated that they mainly used the Dutch national CRC guidelines [18] and/or the national palliative care guidelines [19] to guide their decision-making, and respondents’ familiarity with and use of existing DSS was limited. For example, only four out of 41 oncologists knew the nomogram developed by Fendler et al. [4] ^4^, that predicts the probability of 1-year survival for mCRC patients with inoperable liver metastases after treatment with selective internal radiation therapy (SIRT) (Table 2). One of these oncologists also used this nomogram in clinical practice to inform their own decision-making. Oncologists were not familiar with any other DSS for this setting. Figure 1 provides an overview of the importance specialists give to patient and disease characteristics when devising their treatment advice in the context of mCRC (questionnaire data).

**Table 2:** Oncologists’ familiarity with and use of DSS

		N^a^
Familiarity with DSS (*n* = 31 responders)	Not familiar with any DSS	27
	Familiar with SPICT	0
	Familiar with the nomogram developed by Fendler et al.^b^	4
DSS use in clinical practice (*n* = 21 responders)	Nomogram developed by Fendler et al.^b^	1

*DSS* decision support systems, SPICT Supportive and palliative care indicators tool

^a^Numbers do not add up to 41 due to missing data or because respondents could provide multiple answers

^b^The Fendler et al. nomogram aims to help decision making about whether selective internal radiation therapy (SIRT) is indicated for hepatic metastases of colorectal cancer. Predictions are based on 4 factors, namely: no liver surgery before SIRT, CEA serum level, transaminase toxicity level, and summed computed tomography (CT) size of the largest two liver lesions^4^

### Unmet decision support needs

**Fig. 2:**
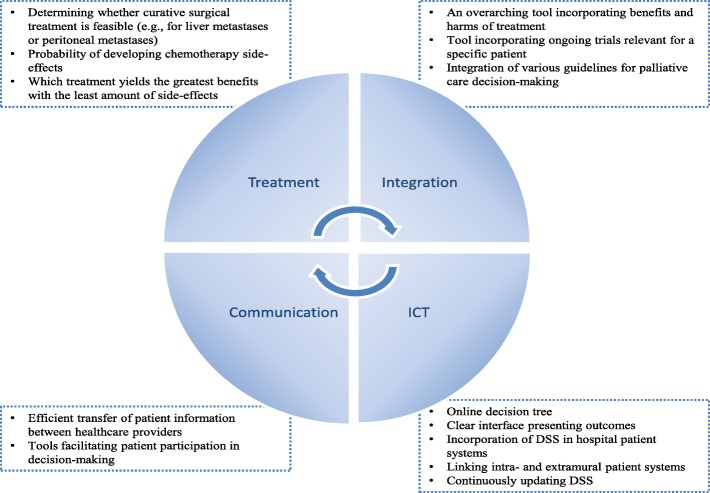
Medical specialists’ unmet decision support needs with regards to mCRC treatment decisions (based on survey and interview data)

In the inventory questionnaire, 28 oncologists indicated that they had unmet needs for decision support, thus requiring the development of a new DSS for mCRC treatment decisions. Thirteen of them wanted a new DSS to support their own decision-making, whilst eight wanted a new DSS to support shared decision-making in clinical practice. Figure 2 provides a summary of the unmet decision support needs oncologists indicated in the inventory questionnaire combined with qualitative data obtained in the interviews.

During the interviews the most recurrent theme was integration. First, integration of the available national guidelines, as currently the colorectal cancer guideline and palliative care guidelines are separate documents located on two different websites. Respondents indicated that with respect to the guidelines, it would be helpful if the guidelines were supplemented with decision trees to facilitate their use in clinical practice. Second, currently available DSS only provide information about one outcome or treatment option, i.e., expected survival with/without a specific chemotherapy regimen or the probability of experiencing a specific side-effect. DSS need to integrate multiple treatment options (e.g., head-to-head comparison of various chemotherapy regimens or surgical vs. radiological treatment) for them to be most effective in clinical practice. Third, tools must present both the benefits and harms of treatment. Preferably, a tool incorporating the first three requirements also features other outcomes, e.g., cost-effectiveness of treatment, and presents a *personalized* overview of ongoing trials for which patients with the selected characteristics are eligible. Ideally, newly developed DSS with good discriminatory ability and good calibration are incorporated in the national guidelines, thereby facilitating their embedding in clinical practice. Another unmet need identified in the interviews, was the need for informatics solutions facilitating communication between intramural and extramural health care providers. General practitioners or community nurses generally know patients for a long time and have better insight in patients’ circumstances. More efficient and thorough exchange of patient information could aid oncologists in hospitals to better estimate patients’ performance status for example. Finally, although oncologists expressed a clear need for DSS, they also conveyed a sense that such tools are not meant to replace clinical judgement, only provide support: *cookbook medicine is not desirable.*

## Discussion

We investigated whether oncologists currently have unmet decision support needs in the context of palliative treatment decision-making for mCRC. We found that oncologists that participated in our inventory questionnaire only knew one DSS for decision-making about palliative care for mCRC, namely the Fendler et al. [4] ^4^ nomogram. However, currently good quality DSS to guide palliative treatment decision-making for mCRC are lacking [12]. Available DSS have several shortcomings that limit their relevance for clinical practice. The methodology used for the development of DSS is not always optimal and often they have not been adequately externally validated or have only undergone narrow validations (e.g., in a single setting or ethnic population) [2, 5]. Additionally, available tools do not meet oncologists’ need for a comprehensive tool. Available DSS do not compare both the pros and cons of multiple treatment options, nor do they present relevant ongoing trials, and additional non-clinical outcomes such as cost-effectiveness of treatment options. Another shortcoming of available DSS is that they do not incorporate all clinically relevant predictors, such as the treatment’s impact on quality of life, the side-effects of treatment, performance status and prognosis. Finally, using informatics solutions facilitating communication between intra- and extramural specialists could also facilitate decision-making. Better exchange of information between healthcare providers contributes to the quality of care in general. However, better information exchange could for example, also improve the accuracy of the assessment of patients’ performance status, which in turn would improve estimates from prediction tools incorporating performance status as a predictor. Given the meager offerings in terms of available DSS, it is perhaps not surprising that respondents have many unmet decision support needs.

Respondents indicated that they currently mainly used the national CRC treatment guidelines [18] and/or the national palliative care guidelines [19] to support their clinical decisions. Even though respondents are confident in the quality of the content of the clinical guidelines, their general nature means they cannot be used to personalize treatment advice. One of the needs expressed was the incorporation of clear decision trees and good quality prediction tools in the online platform where guidelines are located. This would facilitate the embedding of DSS in the clinical decision-making process.

Further, in spite of the growing evidence that some oncogenes (e.g., *KRAS*, *BRAF* or *PIK3CA*), and tumor suppressor genes (e.g., *APC*, *TP53* or *PTEN*) are relevant to prognosis, and could help to personalize treatment selection [13–17], respondents indicated that they currently do not consider genetic markers when pondering about their treatment advice. This is perhaps not surprising as for the mCRC setting more evidence is needed for such markers to be used in daily clinical practice. Given the rapid progress being made in the field of genetic markers and their potential to aid (further) personalization of treatment selection in the future, it is important that such factors are also incorporated in future DSS (once their prognostic value has been established).

If good quality DSS were available, they could help oncologists weigh the benefits and harms of treatment, when deliberating about their mCRC treatment advice. DSS, if they have a suitable patient interface, could also help oncologists to inform their patients about the benefits and harms of treatment. Given these potential benefits, it is pivotal to identify the mCRC treatment decisions for which oncologists have a need for decision support. In our study we focused on this element. A strength of our study is that we had access to all oncologists who are a member of the DCCG. Although the number of respondents is modest, it is likely that the specialists who participated are very experienced oncologists who use and/or feel a need for DSS in their daily practice. As in this study we aimed to inventory the requirements and characteristics of new DSS they need, this makes our sample of oncologists valuable.

Given the complex and multi-faceted nature of palliative treatment decision-making, and the need for a personalized and patient-centered approach, it is somewhat surprising that there are so few good quality DSS available for the mCRC setting. The development of new DSS for the mCRC setting could be of great value to clinical practice. However, development of new DSS meeting specialists’ needs is not straightforward. More research is needed to identify relevant predictors. This is particularly the case for genetic markers to help in the selection of patients for treatment. It is thus unlikely that in the short-term a good quality DSS incorporating genetic markers will be developed. In the meantime, DSS estimating prognosis based on patient and disease characteristics are more feasible and could nonetheless be of added value to clinical practice (for example the mortality calculator proposed by Refro et al. [20]). Further, making DSS available that remain relevant and reliable in the long-term is a key consideration from a methodological standpoint. For any (newly developed) DSS to be useful in clinical practice in the long-term, it is important that the tool is continuously updated. We propose for example, embedding DSS in national patient registries, to facilitate updating as new insights and treatments become available. Finally, although DSS can be of great value in clinical practice and oncologists expressed a clear need for such tools, they also strongly felt that DSS *are* nor *should be* a replacement for their clinical judgement or for deliberation with patients.

## Conclusion

Our sample of oncologists with ample experience treating patients with mCRC indicated that many of their decision support needs are currently not met. Consequently, their treatment advice is almost solely based on the available clinical guidelines. This limits their ability to formulate more personalized treatment advice based on objective estimates of relevant clinical outcomes. New tools integrating multiple treatment options and providing a broad range of clinically relevant outcomes are urgently needed to stimulate personalization of treatment advice and safeguard patient-centered treatment decision-making.

## Abbreviations

CRC: Colorectal cancer
DCCG: Dutch colorectal cancer group
DSS: Decision support systems
mCRC: Metastatic colorectal cancer
SIRT: Selective internal radiation therapy
SPICT: Supportive and palliative care indicators tool
